# Zinc Finger-Homeodomain Transcriptional Factors (ZHDs) in Cucumber (*Cucumis sativus* L.): Identification, Evolution, Expression Profiles, and Function under Abiotic Stresses

**DOI:** 10.3390/ijms25084408

**Published:** 2024-04-17

**Authors:** Yiming Gao, Liyan Zhu, Menghang An, Yaru Wang, Sen Li, Yuming Dong, Songlin Yang, Kexin Shi, Shanshan Fan, Xiaofeng Chen, Huazhong Ren, Xingwang Liu

**Affiliations:** 1 Department of Vegetable Science, College of Horticulture, China Agricultural University, Beijing 100193, China; 2 Sanya Institute of China Agricultural University, Sanya 572025, China; 3 College of Ocean and Agricultural Engineering, Yantai Institute of China Agricultural University, Yantai 264670, China

**Keywords:** ZHD, cucumber, drought stress, VIGS, stomatal movement

## Abstract

Cucumber (*Cucumis sativus* L.) is a globally prevalent and extensively cultivated vegetable whose yield is significantly influenced by various abiotic stresses, including drought, heat, and salinity. Transcription factors, such as zinc finger-homeodomain proteins (ZHDs), a plant-specific subgroup of Homeobox, play a crucial regulatory role in stress resistance. In this study, we identified 13 *CsZHDs* distributed across all six cucumber chromosomes except chromosome 7. Phylogenetic analysis classified these genes into five clades (ZHDI-IV and MIF) with different gene structures but similar conserved motifs. Collinearity analysis revealed that members of clades ZHD III, IV, and MIF experienced amplification through segmental duplication events. Additionally, a closer evolutionary relationship was observed between the ZHDs in *Cucumis sativus* (*C. sativus*) and *Arabidopsis thaliana (A. thaliana)* compared to *Oryza sativa* (*O. sativa*). Quantitative real-time PCR (qRT-PCR) analysis demonstrated the general expression of *CsZHD* genes across all tissues, with notable expression in leaf and flower buds. Moreover, most of the *CsZHDs*, particularly *CsZHD9-11*, exhibited varying responses to drought, heat, and salt stresses. Virus-induced gene silencing (VIGS) experiments highlighted the potential functions of *CsZHD9* and *CsZHD10*, suggesting their positive regulation of stomatal movement and responsiveness to drought stress. In summary, these findings provide a valuable resource for future analysis of potential mechanisms underlying *CsZHD* genes in response to stresses.

## 1. Introduction

Plants frequently encounter a diverse range of environmental challenges, encompassing abiotic stresses such as salinity, drought, and extreme temperature fluctuations, as well as biotic stresses, including physical damage, insect infestations, and pathogen attacks. These stresses exert substantial effects on plant growth, development, yield, and quality [1]. Unlike animals, plants lack mobility, and to survive, they have evolved a comprehensive set of stress–responsive mechanisms. Transcription factors (TFs) play a pivotal regulatory role in mediating responses to these stresses. The activity of TFs is tightly regulated at both the transcript and protein levels, ensuring the successful execution of developmental processes [2]. Among the numerous TF families, those shared by plants and animals are often considered to have particularly important roles. For example, the homeodomain (HD), initially discovered in *Drosophila* [3], stands out as one of the most prevalent protein domains known for its DNA-binding capability in both animals and plants. It is estimated that the HD is present in approximately 110 proteins, constituting almost 5% of the total number of TFs in *Arabidopsis* [4]. Despite the abundance of the HD in plants and the extensive study of some members, such as WOX and HD-ZIP, the functions of numerous other homeobox genes and classes remain largely unexplored. One such example is the Zinc Finger-Homeodomain proteins (ZHDs), which represent a plant-specific subgroup of the HD TFs.

ZHDs are characterized by the concurrent presence of the HD and the zinc finger (ZF) domain [5]. The standard HD is defined by a 60-amino acid sequence, which folds into a three-helix structure, enabling the binding of DNA through the ATTA binding site [6,7]. The ZF domain exhibits significant diversity in both sequence and length and is determined by the arrangement and quantity of cysteine and histidine residues. These residues play a crucial role in binding the zinc ion [8]. MINI ZINC FINGER proteins (MIFs) have been identified as part of the ZHDs and constitute a distinct subfamily distinguished by the exclusive presence of the ZF domain but lacking the HD [9]. The *ZHD* gene family was comprehensively identified in various plants, with instances such as 17 genes in *Arabidopsis* [10], 15 genes in rice [11], 37 genes in wheat [12], 20 genes in Tartary buckwheat [13], 31 genes in Chinese cabbage [14], 22 genes in tomato [15], 11 genes in chilli [16], and 37 genes in cotton [17]. Several ZHDs have been characterized for their crucial roles in diverse developmental processes, such as leaf development [11,18,19], seed longevity [20], hypocotyl growth [21], root hair elongation [22,23], and flower development [24]. In addition, they play a significant role in plant tolerance to various abiotic stresses, including drought [25,26], salt [27], dehydration, and heat [28], as well as biotic stresses like pathogen attacks [29]. In summary, the ZHD family has been identified in numerous plants, each exhibiting distinct potential functions. However, there is currently no report on the function of the ZHD family in Cucurbitaceae.

Cucumber (*Cucumis sativus* L.), a significant species within the Cucurbitaceae family, holds a global presence and serves as a widely cultivated vegetable, playing an important role in China’s vegetable production sector. Despite its importance, the yield and quality traits of cucumber are significantly challenged by drought, heat, and salt stresses [30,31,32]. Previous works indicated that ZHD proteins play a pivotal role in various stress responses by regulating multiple phytohormone pathways in numerous plant species. However, as of now, there is no reported evidence of the function of ZHD proteins in cucumber resistance to stresses.

In this study, we conducted a genome-wide investigation of the 13 *ZHD* genes in cucumber. The analysis included a systematic examination of the evolutionary relationships, chromosomal localization and collinearity, gene structure, conserved protein motifs, and cis-regulatory elements. Additionally, expression patterns of *ZHDs* across different tissues, along with their responses to drought, heat, and salt stresses, were thoroughly analyzed. Interestingly, the results from the transient silencing of *CsZHD9* and *CsZHD10* suggested their potential positive regulation of stomatal movement, indicating their biological functions in drought resistance. These analyses established a robust foundation for subsequent research aimed at elucidating the specific functions of the cucumber ZHDs.

## 2. Results

### 2.1. Systematic Profiles of CsZHD Genes 

**Figure 1:**
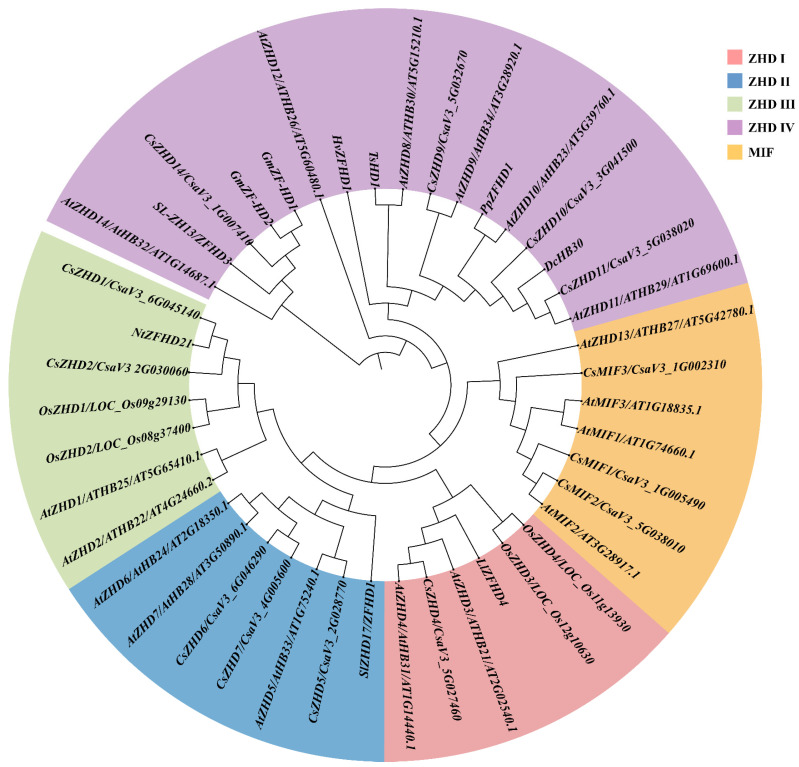
Phylogenetic analysis of the ZHD family. Phylogenetic analysis was conducted on ZHD proteins from *Arabidopsis thaliana* (*At*), *Cucumis sativus* (*Cs*), *Dianthus caryophyllus* (*Dc*), *Glycine max* (*Gm*), *Hordeum vulgare* (*Hv*), *Lilium lancifolium* (*Ll*), *Nicotiana tabacum* (*Nt*), *Oryza sativa* (*Os*), *Prunus persica* (*Pp*), *Solanum lycopersicum* (*Sl*), and *Thellungiella halophile* (*Ts*). The analysis resulted in the categorization of these factors into five distinct clades, designated as ZHDI-IV and MIF.

In the cucumber version 3 genome, a total of 13 CsZHD family members were identified, comprising 10 *CsZHDs* and 3 *CsMIFs*. To investigate the systematic phylogenetic relationships of ZHDs across diverse species, we used the full-length amino acid sequences of all ZHDs from cucumber and Arabidopsis, along with some members with reported functions in rice, tomato, etc. (Detailed information available in Supplemental Table S1), to construct a phylogenetic tree. All CsZHD family members were categorized into five clades (ZHD I-IV and MIF) and named based on the phylogenetic tree (Figure 1). Moreover, we predicted several key characteristics of *CsZHDs* and their encoded proteins (Table 1), encompassing gene ID and position, coding sequence (CDS) length and amino acid (AA) sequence length, as well as protein isoelectric point (pI) and molecular weight (MW). Specifically, AA lengths ranged from 85 (CsMIF2) to 337 (CsZHD6), pI values varied from 6.15 (CsZHD2) to 9.04 (CsMIF2), and MW varied from 9.23 (CsMIF2) to 37.73 kDa (CsZHD6) (Table 1).

### 2.2. Chromosomal Localization and Collinearity Analysis of CsZHD Genes 

**Figure 2:**
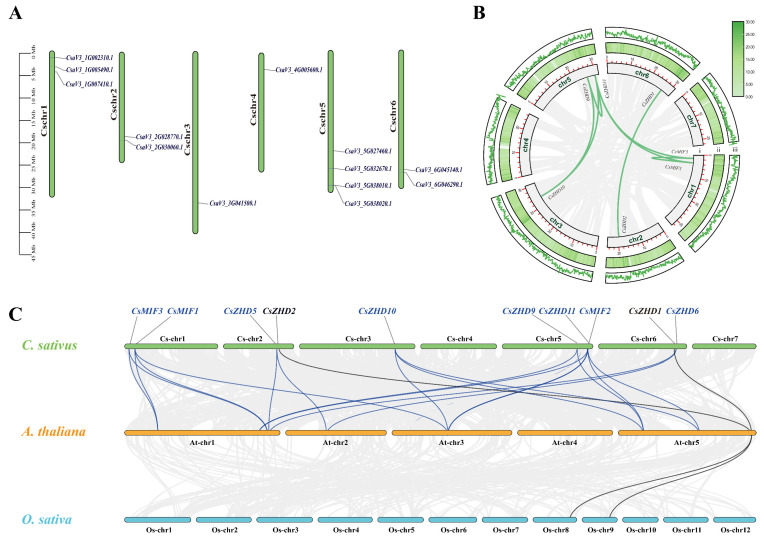
Chromosomal location and collinearity analysis of *CsZHD* genes: (**A**) The locations of *CsZHD* genes are illustrated across six cucumber chromosomes. (**B**) Synteny analysis of *CsZHD* in *C. sativus* genome. Paralogous genes within the cucumber genome are connected by green lines. The number and length of chromosomes (i), gene density heatmap (ii), and gene density bar (iii) are shown. (**C**) Genome-wide synteny analysis of *ZHD* in comparative genomes. Synteny analysis between *C. sativus* and *A. thaliana*, as well as between *C. sativus* and *O. sativa* genomes, is depicted. Blue and black lines represent the orthologous genes.

To examine the physical location of *CsZHD* genes in *C. sativus*, we conducted a chromosomal localization analysis. Figure 2A illustrates the distribution of 13 *CsZHDs* across six cucumber chromosomes, excluding chromosome 7. Notably, there are four genes on chromosome 5, while three and two genes were positioned on chromosomes 1, 2, and 6, respectively. Chromosomes 3 and 4 each contained a single *CsZHD* gene (Figure 2A). Further exploration into the evolutionary dynamics of *CsZHD* genes involved collinearity analysis in the *C. sativus* genome. This analysis revealed seven pairs of segmentally duplicated genes—*CsZHD1* and *CsZHD2*, *CsZHD9* and *CsZHD10*, *CsZHD9* and *CsZHD11*, *CsZHD10* and *CsZHD11*, *CsZHD11* and *CsMIF1*, *CsZHD11* and *CsMIF3*, and *CsMIF1* and *CsMIF3*—indicating the amplification of clade ZHD III, IV and MIF members through segmental duplication events (Figure 2B). To gain deeper insights into the evolutionary relationships of *CsZHD* genes, collinearity analysis was extended to include *A. thaliana* and *O. sativa*. We identified a total of twenty-three gene pairs, with twenty-one pairs by comparing *C. sativus* to *A. thaliana* and two between *C. sativus* and *O. sativa* (Figure 2C). Notably, only *CsZHD1* and *CsZHD2* (indicated in black font) exhibited gene amplification in both *A. thaliana* and *O. sativa*, indicating a closer evolutionary relationship between *C. sativus* and *A. thaliana* in terms of *ZHD* gene evolution compared to *O. sativa*.

### 2.3. Gene Structure and Conserved Motifs of CsZHDs

**Figure 3:**
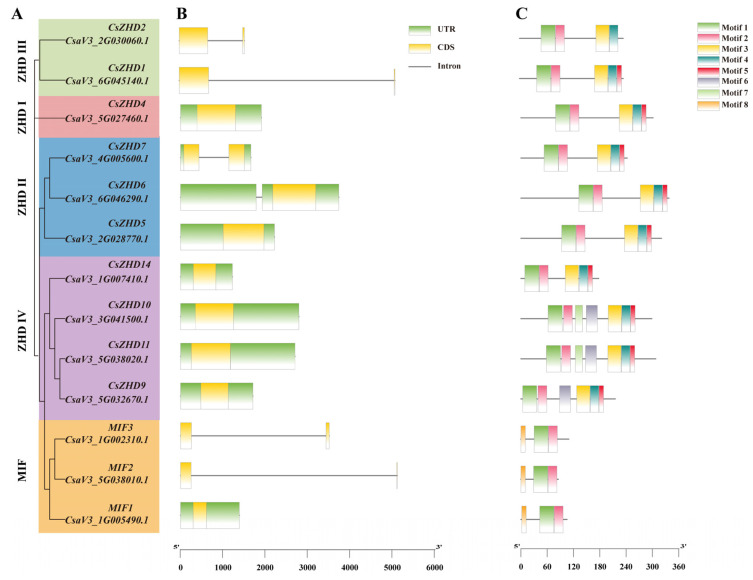
Gene structure and conserved motifs of *CsZHDs*: (**A**) The phylogenetic tree illustrating the relationships among *ZHD* members in cucumber; (**B**) Gene structures of *CsZHDs* are represented, with coding sequences (CDS) depicted by yellow boxes, untranslated regions (UTR) in green, and introns indicated by black lines; (**C**) Eight conserved motifs within CsZHD proteins are highlighted with different colors.

In-depth exploration of the characteristics of *CsZHD* genes involved the investigation of gene structure and sequence attributes of *CsZHDs*. The analysis of gene structure revealed that the majority of genes displayed 5′ and 3′ untranslated regions (UTRs). Particularly, genes in ZHD I and ZHD IV clades exhibited a consistent structural pattern characterized by a single exon and UTRs. In contrast, genes in the ZHD III and MIF clades exhibited a structural composition of two exons and one intron, with the exception of *MIF3* (Figure 3A,B). These findings indicate that genes within the same clade tend to share similar structural characteristics, suggesting a potential functional divergence among different clades.

Additionally, we discovered eight conserved motifs in *CsZHDs*. Motifs 1 and 2 were the ZF-HD dimer domains contained by all detected ZHD proteins (Figure 3C). Motifs 3-54, denoted as the HD, were observed in all ZHD clades except for clade MIF, indicating potential functional distinctions between the four ZHD clades and clade MIF. Motifs 6 and 7 were specific to three members of the clade ZHD IV, while motif 8 was exclusive to all members in the clade MIF. These observations suggest that these motifs may underlie the functional differences across the five distinct clades.

### 2.4. Analysis of Cis-Acting Elements in CsZHD Promotors

**Figure 4:**
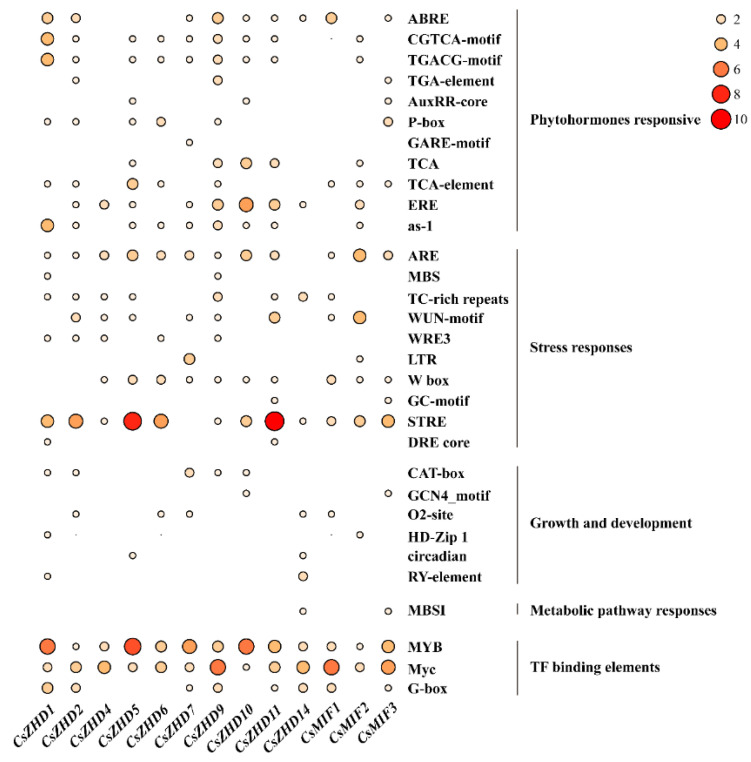
Cis-acting elements in the promoter of cucumber *CsZHD* genes. The size and color of the circles represent the number and types of cis-acting elements present in the promoter regions of *CsZHD* genes.

Numerous studies highlight the pivotal role of the *ZHD* family in plant responses to abiotic stress. Analyzing regulatory elements can offer valuable insights into gene function. In this study, to investigate the regulatory mechanisms of *CsZHD* gene expression, we examined the promoter sequences of all 13 *CsZHDs*. Figure 4 presents the identified major cis-acting elements, with a notable focus on phytohormones such as jasmonate (JA, MeJA(CGTCA/TGACG motifs)), abscisic acid (ABA, ABA-responsive elements (ABREs)), salicylic acid (SA, TCA/TCA-elements), and ethylene (ETH, ERE). This observation suggests potential responses to abiotic stress through various hormonal pathways, particularly evident in *CsZHD1-2* and *CsZHD9-11*. Furthermore, the *CsZHD* promoters harbor ten stress-responsive elements, encompassing drought-responsive MYB binding site (MBS), anaerobic-responsive elements (ARE), wound-responsive elements (WUN motif/WRE3), defense-responsive motifs (TC-rich repeats), heat shock elements (STRE) and elicitor-induced elements (W box). Notably, ARE and STRE were present in all *CsZHD* promoters, except in *CsZHD14* and *CsZHD7*, respectively, indicating potential responsiveness to anaerobic induction and heat shock. Additionally, certain cis-acting elements involved in plant growth and development, such as meristem identity (CAT-box) and the differentiation of mesophyll cells (HD-Zip), were identified. Moreover, the promoters of all *CsZHD* genes contained one or more MYB and Myc binding sites. These findings collectively suggest that *CsZHD* genes may participate in diverse responses to abiotic stresses.

### 2.5. Tissue-Specific Expression Analysis of CsZHD Genes

**Figure 5:**
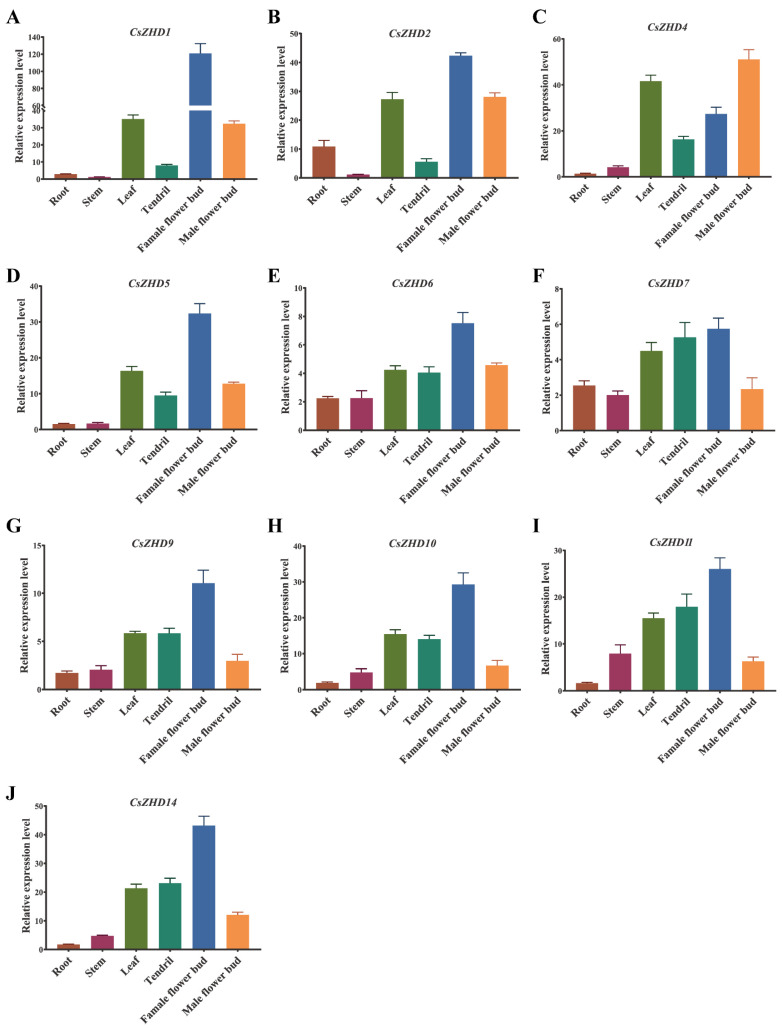
Expression patterns of selected *CsZHD* genes in cucumber tissues. Expression patterns of *CsZHD1* (**A**); *CsZHD2* (**B**); *CsZHD4* (**C**); *CsZHD5* (**D**); *CsZHD6* (**E**); *CsZHD7* (**F**); *CsZHD9* (**G**); *CsZHD10* (**H**); *CsZHD11* (**I**); *CsZHD14* (**J**) in cucumber tissues. Error bars represent standard errors. The *CsUBI* gene was used as an internal standard.

To investigate the *CsZHD* expression patterns, qRT-PCR was used to assess the transcript level of 10 *CsZHDs* (excluding the three *CsMIFs*) in various tissues, including the root, stem, leaf, tendril, female flower bud, and male flower bud, during the reproductive growth stage (Figure 5). Most genes exhibited a similar expression pattern across different tissues, with the highest transcript level detected in the flower buds, particularly in the female flower bud (Figure 5A–J). The majority of genes expressed in the leaf were significantly higher than those in the root, stem, and tendril, including *CsZHD1*, *CsZHD2*, *CsZHD4*, and *CsZHD5* (Figure 5A–D). Notably, *CsZHD6* and *CsZHD7* were highly expressed in the root (Figure 5E,F). Overall, these findings suggest that *CsZHDs* are likely involved in regulating cucumber growth and development across various tissues.

### 2.6. Expression Patterns of CsZHDs under Drought, Heat and Salt Stresses

To comprehensively investigate the expression patterns of *CsZHDs* under abiotic stress, we examined the relative transcript levels of 8 selected *CsZHD* genes under 10% PEG-induced drought, heat at 42 °C, and 150 mmol/L NaCl treatment.

**Figure 6:**
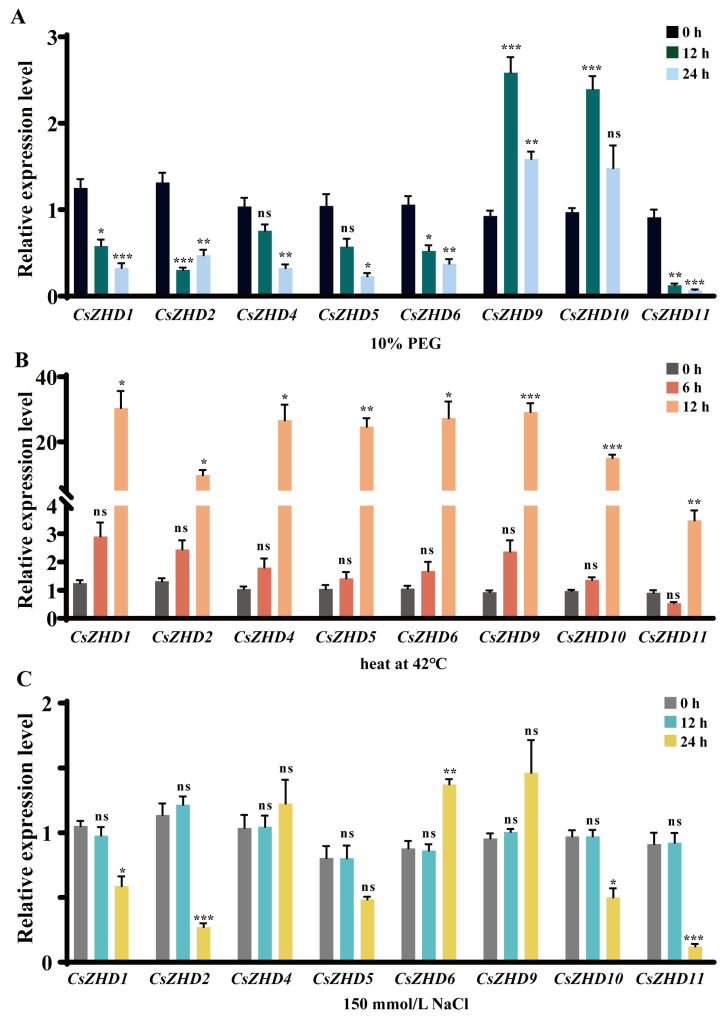
Expression patterns of eight *CsZHDs* under different stress conditions. Expression patterns of eight *CsZHD* genes were analyzed under the following stress treatments: (**A**) 10% PEG; (**B**) heat at 42 °C; (**C**) 150 mmol/L NaCl. Cucumber seedling leaves were collected at 0, 12, and 24 h after treatment with 10% PEG and NaCl, and at 0, 6, and 12 h after heat treatment for expression analysis. Error bars represent standard errors, and significant differences according to the *t*-test are indicated by asterisks (ns, not significant, * *p* < 0.05, ** *p* < 0.01, *** *p* < 0.001).

In the case of the 10% PEG treatment (Figure 6A), two genes, *CsZHD9* and *CsZHD10*, showed upregulation, with approximately a 2.6-fold and 2.5-fold increase in the relative transcript level at 12 h compared to that at 0 h, respectively. Subsequently, their expression levels decreased but remained higher compared to those at 0 h. This suggests a potential positive regulatory role of these two genes in drought stress. Other genes, including *CsZHD1*, *CsZHD4-6*, and *CsZHD11*, exhibited a decreased trend with their lowest expression level at 24 h of PEG treatment. Notably, all eight genes displayed significantly higher relative expression levels after exposure to heat stress at 42 °C compared to that at 0h (Figure 6B). Specifically, the upregulated expression of *CsZHD9* and *CsZHD10* was extremely significant, approximately 30-fold and 20-fold at 12 h compared to that at 0 h, respectively. Five out of the eight genes responded to treatment with 150 mmol/L NaCl, except for *CsZHD4*, *CsZHD5*, and *CsZHD9* (Figure 6C). Among them, four genes were down-regulated following NaCl treatment, including *CsZHD1-2* and *CsZHD10-11*. Notably, *CsZHD2* was significantly down-regulated to only 30% of that at 0 h. These results provide an overview of the differential expression patterns of *CsZHDs* under PEG-induced drought, heat, and salt stresses, highlighting their potential roles in responding to environmental stressors in cucumber.

### 2.7. Silencing of CsZHD9 and CsZHD10 Decreases Drought Tolerance by Regulating Stomatal Movements

**Figure 7:**
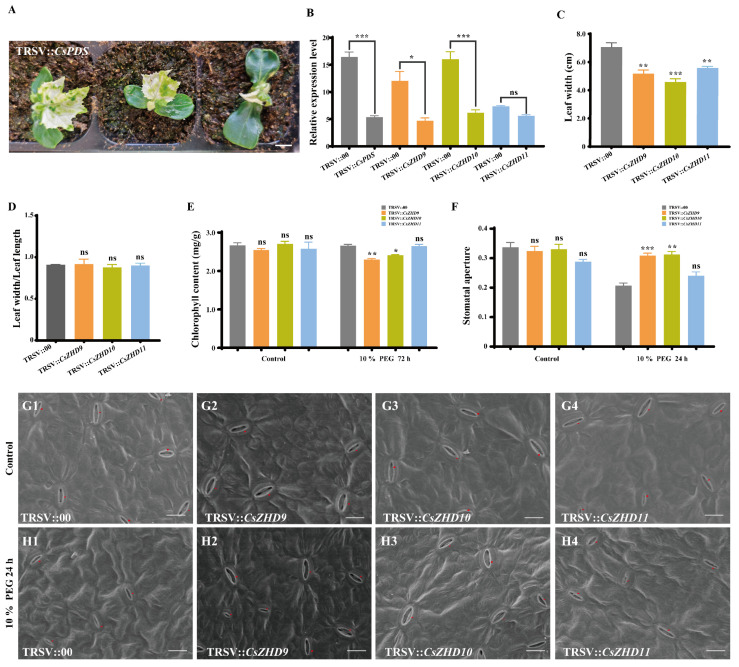
Phenotypic observation and analysis of TRSV::*CsZHD9-11* in drought stress: (**A**) Photo-bleaching phenotype of TRSV::*CsPDS*. Scale bar, 1cm. (**B**) Gene expression levels of *CsZHD9-11* in their respective VIGS-silenced plants. (**C**) The leaf width of the silenced plants. (**D**) The ratios of leaf width and length in the silenced plants. (**E**) Chlorophyll content in the silenced plants. (**F**) Stomatal aperture values. Data measured from 30 stomata. (**G**,**H**) Scanning electron microscopy images of stomata in the silenced plants on the first true leaf in control (**G1**–**G4**) and 24 h under 10% PEG treatment (**H1**–**H4**). The cucumber stomata have been marked with a red color. Scale bar, 20 μm. Error bars represent standard errors, and significant differences according to the *t*-test are indicated by asterisks (ns, not significant, *p < 0.05, ** *p* < 0.01, *** *p* < 0.001).

Our previous results have shown that *CsZHD9*-*11*, which belongs to the clade ZHD IV (Figure 1), of which *CsZHD9* and *CsZHD10* were significantly upregulated by 10% PEG treatment, while *CsZHD11* exhibited a significant downregulation (Figure 6A). To evaluate the functional significance of *CsZHD9-11* in the plant tolerance to PEG-induced drought stress, we conducted tobacco ringspot virus (TRSV)-based gene silencing (VIGS). When TRSV::*CsPDS*-mediated photo-bleaching phenotype was used as a positive control (Figure 7A), we measured the relative expression level of *CsPDS* and *CsZHD9-11* in their respective VIGS plants using qRT-PCR. The results revealed that the relative transcript levels of *CsPDS*, *CsZHD9*, and *CsZHD10* were significantly lower in their respective VIGS plants than those in TRSV::00 (Figure 7B), indicating effective gene silencing. Subsequently, we measured the leaf width, ratios of leaf width and length, and chlorophyll content of silenced plants. The leaf width of TRSV::*CsZHD9-11* was significantly lower than that of TRSV::00 (Figure 7C), and ratios of leaf width and length of TRSV::*CsZHD9-11* showed no significant difference from that of TRSV::00 (Figure 7D), implying that silenced plants exhibited some growth retardation compared to that of TRSV::00. The chlorophyll content of TRSV::*CsZHD9* and TRSV::*CsZHD10* were also significantly lower than that of the TRSV::00 after 72 h under 10% PEG treatment (Figure 7E).

To further understand their response to PEG-induced drought stress, we conducted stomatal observation and measured stomatal aperture values in TRSV::00 and TRSV::*CsZHD9-11*. Under control conditions, no significant differences were observed (Figure 7F,G1–G4). However, 24 h after 10% PEG treatment, most of the stomatal on the leaves of TRSV::00 and TRSV::*CsZHD11* were closed (Figure 7F,H1,H4), whereas those of TRSV::*CsZHD9* and TRSV::*CsZHD10* were still opened (Figure 7F,H2,H3). These results suggest that *CsZHD9* and *CsZHD10* might positively regulate stomatal movement, thereby contributing positively to drought tolerance.

## 3. Discussion

The *ZHD* genes have been acknowledged for their crucial role in governing plant growth and development, along with enhancing adaptability to changes in the external environment, and they have been identified and studied in various species [9,24,33]. In the present study, we identified a total of 13 cucumber ZHDs through BLASTp and phylogenetic analysis (Figure 1). Based on their distinct gene structures, they were categorized into five clades (ZHD I-IV and MIF) (Figure 3). This categorization aligns with findings from prior studies in other species [14,15,34], underscoring the conservation of the *ZHD* family’s evolution across different species.

In order to delve deeper into the evolutionary dynamics and functional aspects of the *ZHD* genes in different species, a gene collinearity analysis was conducted. Gene replication events are predominantly driven by segmental and tandem duplications [35]. Through this analysis, seven segmental duplication events were identified in the clades ZHDIII, ZHDIV, and MIF (Figure 2B). Moreover, the HD, present in all ZHD clades except for the clade MIF, underlines a potential functional disparity between the four ZHD clades and clade MIF (Figure 3C). This divergence in both gene structures and motifs holds the potential for driving functional differentiation among these gene clades.

Additionally, the *CsZHDs* promoters were found to harbor cis-acting elements associated with stress and phytohormone (Figure 4). Previous studies showed an elevation in ABA, SA, and ETH levels in response to abiotic stress, including drought, heat, cold, and salinity [36,37]. MeJA is recognized as a crucial regulatory factor in plant stress responses. Notably, the cis-regulatory elements of ABA, MeJA, and ETH were predominantly present on the promotors of *CsZHD1-2*, *CsZHD7*, and *CsZHD9-11*, suggesting the pivotal roles of these genes in these phytohormone signaling pathways. Consequently, it is anticipated that *CsZHDs* may play a crucial role in both plant development and stress responses.

*AtZHD1*, belonging to the clade ZHD III (Figure 1), has been reported to regulate seed longevity by increasing the content of gibberellin [20]. *OsZHD1*, the homologue of *AtZHD1*, plays a vital role in plant morphogenesis, leaf development, and root meristem activity [11,38,39]. *CsZHD1* and *CsZHD2*, members of the same clade, exhibited high expression levels in the leaf and flower bud (Figure 5A,B), suggesting a conserved function of ZHD III members across different plant species. Moreover, *CsZHD6* and *CsZHD7* displayed high expression in the root (Figure 5E,F), diverging from the other *CsZHD* genes and aligning with the homologous *AtZHD5*, known for its role in promoting root hair elongation [22,23]. *TaZFHD1* has been reported to exhibit a preferential expression pattern at the ‘half, completely emerged’ and ‘half anthesis’ stages during spike development, indicating its potential involvement in wheat anthesis and pollination [40]. Most *CsZHDs* exhibited a similar expression pattern across different tissues, with the highest transcript level observed in the flower bud, particularly in the female flower bud (Figure 5A–J). This observation leads to the speculation that they play a key role in female and male flower development. Overall, these results suggest that the functions of *ZHDs* are relatively conserved yet differentiated among different plants.

In addition, ZHDs not only participate in a variety of plant growth and development processes but also play an important role in the resistance of plants to abiotic stresses, such as *SL-ZH13* [27,41], *HvZFHD1* [28], *PpZFHD1* [42], *DcHB30* [43], *TsHD1* [44], *GmZF-HD1* and *GmZF-HD2* [29] and many other homologues in the clade ZHD IV (Figure 1). Cucumber, a warm-season vegetable, is not cold-resistant and sensitive to high temperatures; it possesses a shallow root system and requires ample water supply. Consequently, it is highly susceptible to drought, high temperature, and salt stresses. In this study, the gene expression level of the *CsZHD* gene family under drought, high temperature, and salt stresses was detected. The qRT-PCR results revealed a high expression level of most *CsZHDs* in the leaf (Figure 5), and their expression was induced by drought, high temperature, and salt stresses (Figure 6). Among them, *CsZHD9* and *CsZHD10* were upregulated after drought stress treatment, suggesting that they seemed to play a positive regulatory role in response to drought stress, while *CsZHD11* from the same clade was downregulated (Figure 6A). Drought stress can trigger ABA production in roots and leaves, inducing leaf stomatal closure and decreasing transpiration to mitigate water evaporation loss [45]. To further investigate the potential role of *CsZHD9-11* in cucumber drought stress resistance, we conducted a VIGS experiment and found that silencing *CsZHD9* and *CsZHD10* reduced the sensitivity of stomata to drought stress and implicated their potential function in cucumber drought stress resistance (Figure 7). These findings suggest that the functions of genes in clade ZHD IV are relatively conserved in response to abiotic stresses in different plants. Moreover, a previous study indicated that pathogens can promote water production between the plant cells, creating a suitable environment for their propagation. In response, plants open stomata, allowing water between cells to evaporate quickly, thus inhibiting pathogen growth [46]. Therefore, stomatal aperture plays a key role not only in plant responses to drought but also in response to other biotic stresses such as pathogen infection. This perspective sheds new light on how *ZHD* genes play an important role in resistance to various stresses. Collectively, these results preliminarily explored the potential function of this gene family in stress resistance and provided a theoretical basis for subsequent in-depth research in cucumber.

## 4. Materials and Methods

### 4.1. Plant Cultivation and Treatment

North China type (Chinese Long) inbred line (CCMC) of Cucumber (*Cucumis sativus* L.) served as the experimental material in this study. Cucumber seedlings were cultivated in a plant incubator under controlled conditions of 25 °C, with a photoperiod of 16-h light and 8-h darkness at the China Agricultural University, Beijing.

For the abiotic stress treatment, the two-true-leaf stage cucumber seedlings were selected and exposed to drought (10% PEG 6000 (*w*/*v*)) [32,47], heat at 42 °C [48,49], and high salinity (150 mmol/L NaCl) [50] stress as described previously. The leaves under PEG and high salinity treatments were collected for 0 h (as control), 12 h, and 24 h, and under heat treatment were collected for 0 h (as control), 6 h, and 12 h, then swiftly frozen with liquid nitrogen, and subsequently stored at −80 °C for subsequent qRT-PCR analysis. Each treatment was conducted with three independent biological replicates.

### 4.2. Phylogenetic Analysis of ZHD Family

To identify potential *ZHD* genes within the *C. sativus* genome, we retrieved amino acid sequences of 17 *AtZHD* genes from the TAIR database (https://www.arabidopsis.org/, accessed on 19 February 2024) and employed them as queries for a BLASTp search against the cucumber (Chinese Long v3 Genome from Cucurbit Genomics Database (CuGenDB), http://cucurbitgenomics.org/blast, accessed on 19 February 2024). We obtained the hidden Markov model (HMM) file associated with the ZF-HD_dimer domain (PF04770) using the Pfam protein family database, accessible at http://pfam.xfam.org/, accessed on 19 February 2024. Validation of the presence of ZF-HD_dimer core sequences involved checks using both PFAM and SMART programs, resulting in the identification of 13 genes containing the ZF-HD domain in the cucumber genome. We further analyzed the characteristics of *CsZHDs*, such as the protein isoelectric point (pI) and molecular weight (MW), using ExPaSy (https://www.expasy.org/, accessed on 19 February 2024). For phylogenetic analysis, ZHD protein sequences from *O. sativa* and *Solanum lycopersicum* (*S. lycopersicum*), etc., were obtained from previous studies [11,28,29,38,39,41,42,43,44,51,52,53], with detailed information available in Supplemental Table S1. To establish evolutionary relationships, we performed multiple sequence alignments of ZHDs in cucumber, *Arabidopsis*, etc., using the ClustalW algorithm within the MEGA 6.0 software. Subsequently, the alignment was utilized to construct phylogenetic trees via the neighbor-joining (NJ) methods, employing 1000 bootstrap repetitions for robustness.

### 4.3. The Analysis of Chromosomal Location and Synteny

The Cucurbit Genomics Database provided information on the *CsZHD* gene locations on chromosomes. TBtools [54] was utilized to map the *CsZHD* genes onto different chromosomes, and the resulting plots were generated based on their physical positions. Syntenic maps for *ZHDs* were created and analyzed using TBtools [54], with visualization performed through Advanced Circos and Multiple Synteny Plot.

### 4.4. The Analysis of Gene Structures, Conserved Motifs, and Cis-Elements

TBtools [54] facilitated gene structure analysis, while the conserved motifs of *CsZHD* proteins were identified using Multiple Expectation Maximization for Motif Elicitation (MEME, https://meme-suite.org/meme/tools/meme, accessed on 21 February 2024). Promoter sequences (defined as 2 kb upstream of the start codon (ATG)) were obtained from NCBI, and all *CsZHD* sequences were submitted to the PlantCARE server (https://bioinformatics.psb.ugent.be/webtools/plantcare/html/, accessed on 22 February 2024) for the analysis of transcriptional response cis-elements. The resulting heatmap was generated by TBtools [54].

### 4.5. RNA Extraction and qRT-PCR

To determine the expression profiles of all ten *CsZHDs* representing the four ZHD clades in different tissues, including root, stem, leaf, tendril, female flower bud, and male flower bud at the reproductive stage, RNA extraction was performed. We extracted the total RNA from the collected tissues using the RNA extraction kit (Huayueyang, Beijing, China). Subsequently, RNA was converted to complementary DNA (cDNA) using a PrimeScript reagent Kit with gDNA Eraser (TaKaRa, Shiga, Japan). The ensuing qRT-PCR analyses were conducted using UltraSYBR Mixture (Low ROX) (Cwbio, Beijing, China) in a CFX384 Real-Time PCR System (ABI QuantStudio 6 Flex, Thermo Fisher, Waltham, MA, USA). We selected eight genes from each of the four ZHD clades to determine their expression patterns under drought, heat, and salt stress treatment. The experimental setup included three biological replicates and three technical replicates for each *CsZHD* gene, along with *CsUBI* (*CsaV3_5G031430*) as an internal reference [55]. Data analysis was performed using the 2^−∆∆Ct^ method [56]. The complete list of primers employed for qRT-PCR is provided in Supplemental Table S2.

### 4.6. VIGS Assay and Phenotypic Observation

To analyze the potential function of *CsZHD9-11*, a modified TRSV-based VIGS assay was performed [57]. Briefly, a 180- to 300-bp specific CDS sequence for the three genes (primers information in Supplemental Table S2) was integrated into pTRSV2 using the SnaBI restriction sites and subsequently transformed into the *Agrobacterium tumefaciens* GV3101. Detailed methods were referred to Feng et al. [58]. A total of 12–16 VIGS plants were generated for each selected gene, with TRSV::00 serving as the negative control and TRSV::*CsPDS* (cucumber phytoene desaturase) as the positive control. The silenced plants were screened by determining the gene expression level of *CsZHD9-11* by qRT-qPCR before physiological analysis. First, the leaf width and the ratios of leaf width and leaf length were measured. Then, 24 h after 10% PEG treatment, the leaf epidermis with the main vein of the first true leaf of each plant was selected for stomatal observations under scanning electron microscopy (SEM), with epidermis with water treatment as the control. The stomatal aperture (the ratio of width and length) was measured and analyzed using ImageJ. Finally, 72 h after 10% PEG, the chlorophyll content of each VIGS plant selected was extracted and measured. Three regions of each VIGS plant were selected as biological replicates and three technical replicates of stomatal aperture observations were conducted for each biological replicate.

## 5. Conclusions

In this study, we identified 13 *CsZHD* members. An analysis of tissue-specific expression revealed a consistent expression pattern for most *CsZHD* genes, with high expression levels observed in the leaf and flower bud. Furthermore, the transcript levels of most *ZHD* genes responded to PEG-induced drought, heat, and salt stresses, particularly *CsZHD9-11*. The transient silencing of *CsZHD9* and *CsZHD10* decreased drought tolerance through the regulation of stomatal movements. These findings establish the groundwork for future exploration of the functions of the *ZHD* members in cucumber.

## Figures and Tables

**Table 1:** Detailed characterization of the cucumber *ZHD* gene family members.

Gene Name	Gene ID	Gene Position		CDS (bp)	AA (aa)	MW (kDa)	pI
		Start	End (+/−)				
*CsZHD1*	*CsaV3_6G045140*	26,710,896	26,715,981 (−)	711	236	25.57	8.83
*CsZHD2*	*CsaV3_2G030060*	19,666,125	19,667,660 (−)	711	236	26.22	6.15
*CsZHD4*	*CsaV3_5G027460*	22,469,732	22,471,636 (−)	906	301	33.59	8.19
*CsZHD5*	*CsaV3_2G028770*	18,872,023	18,874,235 (+)	963	320	34.06	6.52
*CsZHD6*	*CsaV3_6G046290*	27,375,407	27,379,138 (+)	1014	337	37.73	8.79
*CsZHD7*	*CsaV3_4G005600*	3,724,756	3,726,413 (+)	729	242	26.86	8.94
*CsZHD9*	*CsaV3_5G032670*	26,404,212	26,405,916 (−)	648	215	24.26	9.46
*CsZHD10*	*CsaV3_3G041500*	33,868,099	33,870,888 (+)	897	298	31.82	8.49
*CsZHD11*	*CsaV3_5G038020*	30,174,070	30,176,768 (−)	924	307	33.29	8.19
*CsZHD14*	*CsaV3_1G007410*	4,706,303	4,707,523 (+)	534	177	19.34	7.67
*CsMIF1*	*CsaV3_1G005490*	3,579,914	3,581,299 (+)	318	105	11.31	8.86
*CsMIF2*	*CsaV3_5G038010*	30,158,234	30,163,341 (−)	258	85	9.23	9.04
*CsMIF3*	*CsaV3_1G002310*	1,486,596	1,490,100 (+)	330	109	12.16	8.71

## Data Availability

Data are contained within this article and Supplementary Material.

## Supplementary Materials

The following supporting information can be downloaded at https://www.mdpi.com/article/10.3390/ijms25084408/s1.
